# Adherence to pharmacological therapy for hypertension in Portugal: a health professionals focus groups study

**DOI:** 10.1186/s12875-025-02705-4

**Published:** 2025-02-18

**Authors:** Beatriz Rosendo-Silva, João Gonçalves, Filipe Prazeres, Luiz Miguel Santiago, Inês Rosendo

**Affiliations:** 1 Faculty of Medicine, University of Coimbra, Coimbra, Portugal; 2 CINTESIS - Center for Health Technology and Services Research, Faculty of Medicine, University of Porto, Porto, Portugal; 3 Faculty of Health Sciences, University of Beira Interior, Covilhã, Portugal; 4 Family Health Unit Beira Ria, Gafanha da Nazaré, Portugal; 5 FGM University Clinic at FMUC Centre for Health Studies and Research of the University of Coimbra (CEISUC), Coimbra, Portugal; 6 CINTESIS@RISE, Faculty of Medicine, University of Porto, Porto, Portugal; 7 Unidade de Saúde Familiar Coimbra Centro, Coimbra, Portugal

**Keywords:** Medication adherence, Arterial hypertension, Focus Group, Qualitative research, Primary healthcare

## Abstract

**Introduction:**

The perspectives of local healthcare professionals for developing effective strategies to enhance medication adherence in arterial Hypertension as well as its barriers have not yet been explored through qualitative research in Portugal.

**Objectives:**

This study aimed to assess the views of healthcare professionals including general practitioners/family physicians, nurses, and community pharmacists, from Portugal on effective strategies to improve medication adherence in Hypertension, and to identify factors hindering pharmacological adherence.

**Methods and analyses:**

This was a qualitative study with synchronous online focus groups, in which, the participants were general practitioners/family physicians, family nurses, or community pharmacists in Portugal with experience managing patients with Hypertension. They were selected based on age, sex, and geographical region with the number of focus groups determined by theoretical saturation. Recruitment was facilitated through specific mailing lists. Purposive and snowball sampling techniques were employed. Focus group discussions were recorded and transcribed. Two researchers conducted content analyses via MAXQDA^®^2023, applying comparative analysis and reaching consensus. The results are described narratively.

**Results:**

Three focus group discussions revealed a multifaceted approach to improving medication adherence for Hypertension. Key strategies to enhance coordination and communication among healthcare professionals, patients, and caregivers were identified. These included shared informatics software among healthcare professionals; using mobile applications and wearables; health literacy initiatives and patient empowerment; preprepared medication in pillboxes; involving family and the concept of a “family pharmacist”. Participants highlighted barriers to medication adherence such as the lack of communication with patients concerning issues like medication adherence.

**Conclusions:**

This qualitative study outlines strategies to improve medication adherence among patients with Hypertension in Portugal. These involve improving healthcare coordination and communication, patient empowerment, and involving family and “family pharmacists” in supporting adherence. These strategies are based on the insights of healthcare professionals and could be implemented following robust intervention studies.

**Clinical trial number:**

Not applicable.

**Supplementary Information:**

The online version contains supplementary material available at 10.1186/s12875-025-02705-4.

## Introduction

Arterial Hypertension (HTN) is a primary risk factor for cardiovascular disease and death worldwide. Despite the availability of efficacious hypertensive medications [1], blood pressure control remains suboptimal [2, 3], with poor medication adherence (27–40%) being a significant contributor [4, 5]. 

Medication adherence, defined as the extent to which medication intake agrees with recommendations from healthcare providers [1, 6, 7] can be influenced by multiple factors. These factors can be categorized into five dimensions: condition-related factors, therapy-related factors, patient-related factors, healthcare system-related factors, and socio-economic factors [7]. Forgetfulness is a common unintentional contributor to suboptimal adherence [2] and can be affected by specific factors such as mental comorbidities [3].

Enhancing the effectiveness of adherence interventions could significantly increase population health [4]. Although research into medication adherence in HTN has increased in recent years [5] and motivational and complex interventions are considered promising [6, 7], there is a lack of high-quality studies on effective interventions for enhancing medication adherence in HTN [6, 8–11].

Strategies to improve medication adherence focused both on patient and provider factors are recommended [2, 12], and current guidelines suggest that healthcare professionals should communicate the benefits of medication, mitigate side effects, and offer motivational counselling to patients [13]. Moreover, addressing medication adherence through allied healthcare providers seems promising and cost-effective [6]. 

Medication adherence in HTN is influenced by the training, knowledge, and communication skills of healthcare professionals [14], the quality of their relationship with patients, and the provision of positive reinforcement [15]. 

A comprehensive understanding of healthcare professionals’ perspectives is crucial for developing effective strategies to enhance medication adherence as they participate in HTN management, possess unique insights into this issue, and can be a part of the solution.

Although some studies have gathered the opinions of healthcare professionals on barriers to therapy adherence [12, 16–18], no qualitative studies have been conducted in Portugal to explore the views of healthcare professionals, despite the country’s poor control rates of blood pressure (42,5–71,3%) [19–21] and medication adherence (54,6%) [22].

### Objectives

This study aimed to assess the perspectives of healthcare professionals such as General Practitioners/Family Physicians (GPs/FPs), nurses, and community pharmacists, from Portugal regarding the most effective strategies for improving medication adherence in HTN and to identify factors that hinder pharmacological adherence in patients with HTN.

## Methods and analysis

Qualitative research using online, synchronous, focus groups, each targeting six to ten participants over one hour was conducted.

This study adopted a methodology from a previously published, detailed protocol [23]. 

The main moderator was BR-S, a General Practitioner/Family Physicians (GP/FP) with previous experience moderating focus groups, and the moderators’ assistants were IR, a GP/FP who also had a background in moderating focus groups, and JG, a Medical Doctor (MD) who had training from the remaining moderators.

The meeting followed a script (Supplementary material I), and at the end of the focus groups, a summary of the discussion was provided, and participants were allowed to criticise it and add any information.

This study used an online platform, Zoom version 5.13.10, and the focus group sessions were audio recorded and transcribed verbatim.

Before the meetings, the participants signed an informed consent form for the study and a reminder was sent to all participants regarding the upcoming session.

The first focus group confirmed the feasibility of the study and allowed the research team to review the script. The number of focus groups was determined by data saturation [24]. Participants did not receive any form of compensation.

This study followed the established criteria and standards for quality and transparency: the COnsolidated criteria for REporting Qualitative research *(*COREQ) [25] and the Standards for Reporting Qualitative Research [26]. 

### Selection of participants

Participants were GPs/FPs, family nurses, and community pharmacists in Portugal who regularly had contact with patients diagnosed with HTN in a community setting. They were selected based on predefined characteristics such as age, sex, and geographical region (north, central, and south areas of mainland Portugal and the Autonomous regions of Madeira and Azores). To ensure different opinions, each focus group included at least one physician, one nurse, and one pharmacist. The focus group inclusion criteria are presented in Table 1.

**Table 1:** Inclusion criteria of participants

Portuguese healthcare professionals (family physicians, family nurses, and community pharmacists) currently practising in Portugal.	1) involvement in the care of patients with Hypertension in a community setting 2) belonging to distinct geographical regions of Portugal (north, central, and south areas of mainland Portugal and the Autonomous regions of Madeira and Azores) 3) different ages (no age limit applied) 4) both sex representation

The authors promoted the study through specific mailing lists of healthcare professionals. Healthcare professionals who replied by showing interest in participating received more details about the study. If selected to participate based on the inclusion criteria, they received a copy of the participant consent form via email. During the selection of participants for the focus groups, participants’ availability for each meeting date and time was given priority. After generating three pools of potential participants based on their profession, a random number generator was used to select them to minimise selection bias.

However, because the number of volunteers was small, snowball recruitment [27] was also used with participants who agreed to participate.

To achieve maximum sample diversity, a purposive sampling [28] method was used.

The focus group interviews took place in December 2023.

No financial benefit was provided to the participants.

### Data collection

Data were collected during the focus groups and recorded on the Zoom online platform. The facilitator BR-S promoted the debate using non–directive moderation with open-ended questions from the script to ensure self–containment regarding the involvement in the group [27]. 

Data transcripts were not returned to participants due to their large extent and to ensure confidentiality.

Additional participant sociodemographic data were collected in an online form from Google Forms at the end of the focus group.

### Data analysis

Data analysis was performed using proper qualitative software, MAXQDA^®^ 2023.

Two independent researchers (BR-S and JG) performed content analysis through comparative analysis and posterior consensus.

Initially, they set the level of abstraction as higher and constructed a coding tree with the main themes and categories, which mainly corresponded to the script questions that were built based on prior research [23, 29]. First, deductive analysis was guided by these predefined themes and categories and data was labelled. Then, new categories and subcategories emerged from codes derived from meaningful units of the transcripts through inductive analyses and the level of abstraction was resettled [30–32]. The categories and subcategories were revised to check category overlaps to merge or to divide into subcategories [29]. 

A third researcher (IR) reviewed the analysis.

The findings were displayed mainly in tables with narrative descriptions, including illustrative quotes, which omit identifying details to maintain confidentiality. When presenting the results, the barriers to medication adherence were organised according to the WHO Multidimensional Adherence Model [4]. 

These results were then compared with the 15 most pertinent strategies identified from the transcript analysis.

Participants did not give direct feedback on the findings.

## Results

Three focus groups were conducted, amounting to a sample of 22 participants. Eight individuals withdrew from the study and did not attend the meetings, only two of them justified having other commitments.

Participants were mainly female, with a mean age of 42.23 ± 11.11 years (range: 27–67). Among them, four (18.2%) lived in northern Portugal, 15 (68.3%) lived in the central region, one lived in Lisbon and Vale do Tejo, one lived in Algarve, and one lived in thz Autonomous Region of Madeira.

The sample included nine GPs/FPs, six nurses, and seven pharmacists. Academic qualifications ranged from undergraduate to doctoral degrees, with the majority (63.6%) holding a master’s degree.

Nine participants (40.9%) had previously participated in a focus group study.

The characteristics of the participants are detailed in Table 2.

**Table 2:** Participants’ characteristics

Variables		*n* = 22	%
Age	42,23 ± 11,11 (27–67) years		
Sex	Female	17	77,27
	Male	5	22,72
Area of Residence	North	7	77,27
	Centre	15	68,18
	Lisbon and Vale do Tejo Algarve Autonomous Regions of Madeira	1 1 1	4,55 4,55 4,55
Profession	GP/FP	9	40,91
	Nurse	6	27,27
	Pharmacists	7	31,82
Academic qualifications	Graduation	6	27,27
	Master’s degree	14	63,64
	PhD	2	9,09
Previously taken part in a focus group	Yes	9	40,91
	No	13	59,09

### Definition of adherence to pharmacological therapy

Regarding the definition of adherence to therapy, participants stated that it involves patients taking the prescribed medication, in agreement with their physician while emphasizing the importance of the relationship between them.

The patient takes their medicines as agreed with their physician, based on a shared decision.

Healthcare professionals also believe that educating and raising awareness of patients’ chronic diseases and their therapy is crucial for medication adherence.

*“I would say that the first thing I value is the patient being aware that he has a chronic illness; that’s the first step. Adherence is when they are truly aware that they need to take action to control their chronic illness.”*

### Forgetfulness in medication adherence

Participants identified the main reasons young adults forget to take medicines, such as having busier routines, leaving the house in a rush, and discontinuing their medication once they achieve controlled BP levels or because they have no symptoms (Table 3). Regarding older adults, healthcare professionals mentioned different reasons, such as cognitive impairment and logistical failures due to dependence on family members or caregivers. Participants also pointed out that older adults easily forget to take medication if they are in polypharmacy, particularly when there are adjustments to their medication.

Irrespective of age group, lack of an established routine seems to contribute to forgetfulness to medication adherence.

**Table 3:** Reasons for forgetting to take medication

**YOUNGER ADULTS**	
Being in a rush and busier routines	“For younger individuals, the issue is the rush” “(…) [The younger adults] find it challenging to integrate this routine and habit because their schedules are much more demanding (…), leading to forgetfulness, not because they don’t value it (…)”
Controlled BP/asymptomatic disease	“Their blood pressure gets controlled, and they discontinue their medication. They experience no symptoms and, consequently, feel no need to continue taking their medication (…)” “(…) If they do not measure their blood pressure, they do not see any significant improvement that would make them think they need to continue taking it [the medication] to feel better.”
**OLDER ADULTS**	
Memory lapses	“For elderly individuals, it’s memory loss, isn’t it?” “They (…) get distracted by something else, and completely forget.” “(…) and when there are early signs of dementia, they sometimes cannot follow any treatment regimen properly, and we notice that they become more confused than usual.”
Dependence on others	“They depend on others and sometimes miss doses, often due to forgetting to purchase the medication. Frequently, there is no proactive management of medication stock, which sometimes leads to missed doses.”
Polypharmacy	“(…) I believe it also relates to the fact that patients have multiple medications to take, particularly those on polypharmacy regimens. When there are changes in their medication, it causes significant confusion, and there are people that prefer to stop taking their medication until they can talk to the physician or the healthcare professional.” “(…) one of the common barriers that patients mention regarding taking their medicines is: ‘Doctor, I take so many pills.’ They inevitably end up forgetting to take some.”
**IRRESPECTIVE OF AGE GROUP**	
Lack of routine	“(…) the main reason for forgetting is the lack of routine (…)” “(…) do not have a routine for measuring [Blood Pressure] and taking medication (…)

### Side effects and strategies to mitigate side effects

The most frequently mentioned side effects were increased urinary output due to diuretics and coughing due to angiotensin-converting enzyme inhibitors (ACE inhibitors).

Adverse effects related to beta-blocker therapy were also discussed, such as possible changes in heart rate, reduced libido, and erectile dysfunction. Additionally, healthcare professionals mentioned dizziness, headache, and peripheral oedema, the latter being caused by calcium channel blocker (CCB) therapy (Table 4).

To mitigate side effects, participants recommended regular blood pressure monitoring by pharmacists and communication improvements between pharmacists and GPs/FPs.

In Primary Health Care (PHC), health professionals should verify that the patient has no relative or absolute contraindications to the prescribed medication. Additionally, they should tailor the medication choice according to each patient’s characteristics and avoid making major adjustments to the medication each time it is updated.

Participants also recommended improving the clinical approach by conducting more comprehensive medical histories and extending the expected length of each consultation so that health professionals can educate the patient and clarify any doubts (Table 4).

Furthermore, whether patients should be educated about potential side effects has been debated. One participant believed that such actions could have the opposite effect, leading to medication avoidance.

**Table 4:** Side effects and strategies to mitigate them

**Side effects**	
“(…) they [the patients] have to go to the toilet frequently, which is very inconvenient.” “(…) the cough associated with ACE inhibitors is a well-known side effect, but it is a long-term effect and sometimes difficult to assess.” “(…) they value issues like swelling in the legs” “(…) with beta-blockers, they may experience changes in heart rate that are symptomatic.” “(…) dizziness and headaches, in the initial phase (…)” “(…) sometimes they mention issues related to libido and sexual performance.”	
**Strategies to Mitigate Side Effects**	
Improving Patient Approach	“It doesn’t make sense to take diuretics at night because the patient might wake up.” “(…) adjusting the medication so that the effects have no impact on the person’s life.” “Begin with low doses and adjust according to the response. Avoid combining medications right from the start; follow a step-by-step approach.” “(…) with a well-conducted clinical history, with the necessary time, which we don’t always have.” “ (…) informatic applications to check interactions”
Patient education on side effects	“(…) informing them [the patients] of potential side effects so that they are aware and mentally prepared about them.” “I’ don’t agree with this approach. (…) because if we tell them, nearly all of them will definitely experience side effects. If we warn them that they may have increased urinary output, then they won’t take the medication.”
Monitoring BP at pharmacies	“(…) When dealing with older individuals who may be afraid, we suggest (…) checking your blood pressure. Visit us a few times during the week and we will see how you react [to the new prescription].’
Pharmacist-GP/FP Communication	“(…) actively conducting medication follow-up and sending such information to the doctor, improving our communication with the GP/FP.”

### Barriers to pharmacological adherence in HTN

Participants identified several barriers to medication adherence (Table 5).

It was highlighted that the absence of symptoms in HTN often leads to a lack of concern about proper medication adherence and many patients discontinue their medication once their blood pressure improves. On the other hand, if patients experience some discomfort like lethargy due to low BP levels or medication-related side effects, they might stop taking their medication.

Healthcare professionals have argued that patients on polypharmacy overlook the importance of medication adherence because they have too many pills to take; and have difficulty sharing personal misconceptions about medication at appointments. Previous medication experiences, and the influence of others when they are misadvised to stop medication, also decrease therapeutic adherence.

Regarding healthcare system-related barriers, running out of medication without a new prescription, lack of effective doctor-patient communication about medication adherence, and a prescription schedule that does not align with the patient’s routine were identified as reasons for missing medication.

In addition, medication costs were identified as an important barrier to medication adherence.

**Table 5:** Barriers to medication adherence

CONDITION RELATED FACTORS	
“Silent” Disease	“(…) the fact that many of them are asymptomatic and do not understand what hypertension is, makes them fail to recognise the importance of adhering to treatment.” “(…) the effect [of the medication] is not felt (…) if I have pain, I take an analgesic, and the pain disappears, but if I take a medication for hypertension, I do not feel whether I am better or worse.”
Polypharmacy	“(…) they stop taking some medications when they have many to take.” “(…) when there are too many pills, there are always some [pills] that they end up not taking.”
THERAPY-RELATED FACTORS	
Symptoms of hypotension	“(…) when blood pressure drops too much, they do not feel well and stop the medication (…) they end up feeling some discomfort, lack of energy, weakness, lethargy, and they associate these symptoms with the medication, which is one of the reasons they tell us.”
Side effects	“(…) often side effects lead to poor adherence, particularly if it’s a diuretic and the patient must go out. They easily skip the medication because they need to manage these issues.” “(…) in older adults, it is often related to the side effects.”
Past experiences	“(…) past experiences with medication (…)”
Not aligned with routine	“(…) our prescription may not align with the patient’s routine. If the person works shifts or consistently forgets [to take the medication] at dinner, or if they tell me that they can’t manage it at dinner, perhaps I would prefer they take it in the morning rather than not take it or forget it a lot.”
PATIENT-RELATED FACTORS	
Blood Pressure Control Beliefs	“(…) when they notice blood pressure is improving, they stop taking the medication.” “(…) they often believe that they are no longer hypertensive patients.”
Personal Beliefs	“(…) beliefs about diseases and life, and the notion of self-care that people have, even regarding their sense of responsibility.” “Popular beliefs, alternative medicines, and the difficulty in sharing it during an appointment, meaning they [patients] agree with the prescribed medication only because they do not feel comfortable enough to say they are following other methods.”
Influence of others	“(…) and the influence of others – ‘Oh, you’re taking that? That’s bad for you,’ or ‘It doesn’t matter if you miss a day or two. The treatment will still work.”
HEALTH CARE SYSTEM FACTORS	
Lack of communication between patient and physician	“(…) if we do not actively talk about it, we often assume that they are taking it [the medication], and if this is not openly discussed, there may be missed doses that are not mentioned during the consultation.” “(…) one of the main reasons for forgetfulness is the lack of understanding of the therapy. We believe our communication is effective, but that is not always like that.” “(…) it’s the failure to ask during appointments whether there is adherence to the treatment.”
Running Out of Medication	“Because it [the medication] ran out and they didn’t have a consultation, they were waiting for an appointment to get a prescription.” “Difficulty in accessing a prescription may also lead the patient to wait for the consultation and, even if they run out of medication before [the appointment], they wait for the consultation to request a renewal.”
SOCIO-ECONOMIC FACTORS	
Medication Cost	“Due to their low pension, they tell us they cannot afford the pills” “(…) patients do not take the medication every day because they are trying to save money.”

### Role of technology and family members in medication adherence

Participants agreed that strategies that include technology may have a role in medication adherence (Table 6), but they argued that the oldest patients resist adopting technologies like the official national service healthcare App - *SNS24* - mostly because they do not know how to use them or they are not capable of using them.

*“(…) we encourage them to install the SNS24 app to access their prescriptions*,* but often they do not want to*.”

*“Most of our oldest patients do not use apps.”*

It was stated that families usually assist and support patients, motivating and ensuring medication adherence.

“*They even encourage each other to adhere to their therapies.*”

“*They prepare the medication and organise the pillboxe*s.”

“*When there are difficulties in going to the health centre or pharmacy by themselves, [the family] pick up the medications and prescriptions on their behalf.”*

However, family members can also be absent or not physically capable of helping or acting as disruptive elements in treatment adherence, particularly when more than one family member has HTN, potentially leading to confusion regarding the appropriate therapeutic regimen.

“*Often, families are not present*.”

“*Nowadays, older adults are taking care of other older adults, isn’t it? Sometimes it’s a mother who is 90 years old, and the daughter is already 70 or 60*.”

“*(…) others are completely the opposite and should take different medications because if not, they completely confuse each other*.”

It was also noted that patients sometimes refuse help from their families and that patients should be included in the decision regarding whether family support should be involved in medication management.

“*When the family tries to help, (…) the older adult reacts poorly because they feel that they are being told they are not capable of managing their medication*.”

*“(…) it must be done with the agreement of the older adult or the more dependent individual that this is necessary and that they accept that this person [family member] assumes the role [of medication management]*,* in order to avoid conflicts of interest.”*

### Strategies to improve adherence to antihypertensive medication

According to the participants of the focus groups, the key strategy was to improve the coordination and communication between health services among themselves and caregivers as they should be a team and share information about medication adherence.

Engaging caregivers or family members to come to appointments and help manage medication and providing access to the national electronic prescription platform called *Prescrição Electrónica Médica (PEM)* to pharmacists would enhance the quality of communication. Developing home visits to patients to ensure medication adherence and promoting health literacy through public health education in schools and on television were also proposed as coordinated measures to improve medication adherence in HTN.

Regarding technology, participants highlighted the need to incorporate technology into patients’ daily lives, like using mobile phones to set alarms to remind them to take their medication and renew their prescriptions. The development of watches to measure blood pressure was also suggested. Healthcare professionals acknowledged the value of mobile applications (apps) designed to remind users to take their medication and to measure and record their blood pressure and argued that health systems could play a more active role in promoting these applications. In this context, the participants stated that it would be beneficial to develop an app or platform that connects the patient with the GP/FP, community pharmacist, and family nurse (Table 6). The idea of patients wearing watches that enable blood pressure measurements was seen as promising.

At the HPC Centres, participants suggested extending the appointments for managing HTN to provide more time for educating patients and addressing their concerns. They also recommended updating the PEM to simplify the process of medication adjustments and that GPs/FPs reduce therapy complexity by decreasing the number of pills the patient should take. In addition, the participants proposed that the PEM system allows detailed prescriptions to be sent through patients’ mobile phones or that a therapeutic guide with the purpose of each drug in an understandable language should be provided when delivering the prescription.

At the pharmacies, it was mentioned that it would be useful to involve pharmacists in medication management and allow them to check if a patient has the necessary number of pill boxes and allow pharmacists to renew their chronic medications in collaboration with the GP/FP. Healthcare professionals spoke about the need to create a “family pharmacy” and have alerts to pharmacists when medication adjustments are made.

To assist patients in adhering to their medication regimen, participants recommended placing the medication in a visible and accessible location linked to a morning routine to prevent forgetfulness. They emphasised the importance of patients being aware of their chronic disease, the need for chronic medication, the consequences of missing medication, and involving them in disease management, such as selecting the most suitable schedule for taking the medication.

The use of a preprepared pillbox with distinct compartments for each day of the week to reduce the risk of missing medication doses was also proposed.

**Table 6:** Strategies for enhancing hypertension treatment adherence regarding Healthcare services

**BETTER COORDINATION OF HEALTH SERVICES**	
Communication system among health professionals	“(…) all strategies that improve communication among healthcare professionals (community pharmacists, family nurses, and GP/FP) and the patient will always enhance everything.” “The ability to collaborate and have a communication system is very important because patients interact with many health professionals, but there is no interconnection of all this information.” “(…) improving communication between health professionals is urgent.” “In my opinion, the pharmacist should have direct access to PEM.” “It would be important to standardise the information technology systems of professionals”
Caregivers’ engagement	“(…) communication between caregivers and the family nurses and GP/FP units is essential.” “(…) a family member would come along with the patient to help manage the medication (…)” “(…) engaging the family in the preparation of the medication, reviewing and organising it.”
Home Visit	“(…) a home visit conducted by different health professionals”
Technology to help patients	“Reminders to visit the pharmacy to purchase new medicines.” “Apps that ensure that people do not forget, such as reminders to take medication at a specific time and allowing the therapeutic schedule to be included in the app.” “I think an app that facilitates communication between healthcare professionals (the doctor, the family member, the patient, the pharmacist, the nurse), where it would be possible to note down measurements, record medication changes, and that also has an alarm feature would be useful. (…)” “(…) [In the future] the patient could have a watch that could do it [measuring blood pressure].”
Globally increasing health literacy	“(…) national or local campaigns (…) will improve health literacy.” “It could be done through national television, like an advertisement on the importance of hypertensive medication (…) or starting in schools, focusing on prevention and information.”
Reduction of Medication Cost	“(…) reduction of medication cost to enhance medication adherence.”
**PRIMARY HEALTH CARE**	
Extend Consultations	“(…) the hypertensive appointment should be a time where the healthcare professional has more time available to empower the patient.”
Updating PEM	“PEM updates could include an alert notifying the dose has been changed.” “PEM could send a proper guide for the mobile instead of just sending strange codes.” “(…) Changing the dose of medication while using PEM should be easier and automatic (…)”
Reducing Therapy Complexity	“(…) monotherapy enhances adherence to the therapeutic regimen.” “(…) the more complex the regimen, the lower the patient’s adherence will be.”
Therapeutic guide	“(…) patients should be given a therapeutic guide, which is very important (…)” “I write in the therapeutic guide what is the purpose of each pill in an understandable language.”
Patient Empowerment and disease Awareness	“(…) having awareness of the disease, that is the most important thing (…)” “(…) explain that BP control is closely related to taking the medication.” “(…) involve the patient, (…) explaining the impact of not taking the medication properly and trying to negotiate with the patient.” “ask the patient if they rather take the medication in the morning or at dinner”
**COMMUNITY PHARMACIES**	
Involvement in Adherence Management	“(…) pharmacists should be involved in managing whether a person is purchasing fewer boxes than expected.” “(…) chronic medication renewal at a pharmacy would be important, meaning it would not require a prescription. (…) and whenever there’s a change [in the prescription], the pharmacy must have access to that information.” “The renewal of chronic medication following strict *guidelines* in coordination with PHC Centres”
Family Pharmacy	“It’s also important to have almost a family pharmacy.”
**PRIMARY HEALTH CARE/ COMMUNITY PHARMACIES**	
Medication preparation	“(…) preparing medication at the PHC Centre, so the patient leaves with a week’s supply in a pillbox when there isn’t a family available to do it.” “(…) boxes for older adults with preprepared doses in a central pharmacy.”
Advise patients to place the pills strategically	“(…) if the pills were taken right after getting out of bed and if people had a small bottle on their bedside table, they would forget them less.” “I usually suggest placing it where there is less chance of forgetting.” “If the patient takes the medication in the morning before breakfast and uses the microwave every morning, place the tablets next to the microwave.”

## Discussion

### Main findings

The focus group discussions revealed a multifaceted approach to improving medication adherence in HTN in Portugal.

The primary emphasis centred on enhancing coordination and communication among healthcare professionals, patients, and caregivers, recognising the importance of information sharing through integrated informatics software between GPs/FPs, family nurses, and community pharmacists. Participants advocated for family member’s involvement in consultations, the development of a mobile app to connect patients or caregivers with their healthcare providers, and the promotion of health literacy through public education initiatives. The integration of technology into patients’ daily routines, such as mobile phone reminders to take and purchase medication alongside remote blood pressure monitoring, was highlighted as a promising approach.

Several important recommendations emerged from this qualitative study: patient empowerment, provision of a comprehensible therapeutic guide with a simplified regimen, preprepared medication in pillboxes, reduced medication costs, and a “family pharmacist” to help in medication management.

Regarding barriers to medication adherence, participants identified diverse categories: condition-related and therapy-related barriers, the influence of others and patients’ beliefs, and the lack of communication with patients concerning issues like medication adherence.

### Strengths and limitations of this study

This focus group study explored insights and views from Portuguese healthcare professionals, which were shaped by social interaction and facilitated a more authentic collection of their ideas on an understudied subject [32, 33]. 

The moderators’ expertise in qualitative research enabled attentive listening and allowed them to remain reflexive throughout the meetings. Furthermore, a validated script was used to ensure the meeting’s quality.

Participants had experience in the research topic, enhancing their willingness to share their insights, and they had a broad age range (27–67 years), which facilitated a diverse range of opinions.

Although there was at least one healthcare professional from each region of the country, most participants belonged to the central area of Portugal.

Despite having the chance to provide feedback at the end of each meeting summary, participants did not have access to the final analysis and two participants were known to the main facilitator, which may have introduced desirability bias.

The verbatim translation into English may have resulted in cultural nuances or loss of meaning.

The findings from these focus groups are specific to the Portuguese healthcare system, as intended, and may have limited generalisability to other contexts.

### Discussion of results and comparison with existing literature

Our study found that improving the care coordination between family nurses, community pharmacists, and GPs/FPs in Portugal can improve medication adherence among patients with HTN. This should include enhancing communication between these healthcare professionals through shared informatics software, enabling them to verify dispensed prescriptions, access current medication modifications, and monitor if the medication is being correctly taken. These specific measures have not been identified in other studies, potentially due to their unique relevance to Portugal’s healthcare system.

Including a caregiver or family member in HTN consultations to help with medication management aligns with the existing research, suggests medication counselling [34] or update on regularly medication [35] for caregivers and states that the family’s role in medication adherence is fundamental in encouraging medication adherence [36]. 

Although not universally accessible, technology has been previously proposed as a tool to improve medication adherence [37, 38]. Some interventions utilising apps have been tested in RCTs, though the predominant focus was to improve health literacy through educational content [39, 40]. Our study highlighted the need for coordinated health literacy.

We identified some studies featuring apps that included patient reminders to take medication, BP recording capabilities; healthcare providers or caregiver notifications for abnormal BP readings, and enabled healthcare providers to check the uploaded data and give feedback to their patients [41–43]. Our participants added that technological reminders should include medication purchase alerts and that apps should facilitate communication with healthcare teams. The proposal of wearables like a watch to monitor BP remotely corroborates previous findings on remote patient monitoring improving HTN care [44].

The recommendation to extend the length of appointments to allow healthcare professionals sufficient time for patient empowerment in HTN management may reflect the perceived barrier that physicians do not discuss issues like medication adherence [45] This is consistent with other Portuguese studies indicating patients feel that doctors conduct consultations at a fast pace [37]. Additionally, this aligns with literature supporting self-management interventions’ beneficial effects on older adults with HTN [45]. 

Consistent with other studies reporting therapeutic complexity as a barrier to medication adherence in cardiovascular patients [45], our participants advocated for simplified therapeutic regimens – a crucial consideration for health professionals.

Other strategies to improve adherence to antihypertensive medication were pharmacists’ active involvement in medication management, particularly addressing side effects and establishing a “family pharmacist” role to be coordinated with PHC care. Although strategies addressing community pharmacists have been developed with some of them in collaboration with GP/FP [46], only one randomised controlled trial (RCT) with a multi-collaborative initiative has been conducted in Portugal [47] which did not show effectiveness and did not apply this specific concept.

This study indicated that medication preparation services can be valuable for improving medication adherence among older adults with HTN. Similar ideas have been suggested [37] although evidence for such interventions remains limited [48].

Regarding barriers to medication adherence in HTN, although they were mainly described in a recent systematic review [36], our findings present a specific selection reflecting Portuguese healthcare providers’ perspectives.

### Implications for research and clinical practice

A larger number of GPs/FPs, community pharmacists, and family nurses from PT will, in a subsequent phase, be surveyed about the best strategies for improving medication adherence using a questionnaire developed from these findings as a framework. This qualitative study is part of a broader research that also examines the perspectives of Portuguese patients with HTN. Following the triangulation of these perspectives, the best strategies to enhance medication adherence in HTN should be evaluated through an RCT in Portugal.

This qualitative study recommends several strategies to improve medication adherence in HTN focusing on healthcare professionals’ perspectives and addressing barriers to medication adherence in HTN within the Portuguese context.

This study was conducted with a local population and offers practical recommendations to the healthcare system and providers that may enhance the quality of HTN care and help address suboptimal rates of medication adherence and HTN control in Portugal.

## Conclusion

This qualitative study addresses a significant gap in patient care by outlining strategies to improve medication adherence among patients with Hypertension in Portugal. These strategies involve improving healthcare coordination and communication, patient empowerment, and involving family and “family pharmacists” in supporting adherence. They are based on the insights of healthcare professionals and could be implemented following robust intervention studies.

## Electronic supplementary material

Below is the link to the electronic supplementary material.

Supplementary Material 1

## Data Availability

Not applicable. As stated in the protocol of this study, the research team will keep all confidential data for 5 years, storing it exclusively on password-protected computers that are only accessible to team members.
