# Supplementary material for: Adherence to pharmacological therapy for hypertension in Portugal: a health professionals focus groups study
Source: BMC Prim Care. 2025 Feb 18;26:44. doi: 10.1186/s12875-025-02705-4 (PMC11834486; doi:10.1186/s12875-025-02705-4)
Supplement: Supplementary file 1 — Supplementary Material 1 [file 12875_2025_2705_MOESM1_ESM.docx]

**Supplementary material I – Script Used During the Focus Group Sessions**

**Acknowledgement:**
Thank you for agreeing to participate in this meeting aimed at gathering your insights. We appreciate your interest in taking part in this meeting.

**Introduction:**
Introduction of the moderator and the research team.
This study is being conducted as part of a research project, and this focus group aims to gather information regarding the barriers to medication adherence for Hypertension (HTN).
This session will last approximately 1 hour and 15 minutes. There will be a moderator responsible for asking questions and guiding the discussion, as well as an assistant moderator who will take notes during the session.
We need your opinions to help us gain different perspectives on this topic. We encourage you to share your thoughts and provide your honest opinions.

**Guidelines for the Session:**

1. We want you to speak and debate the topics. We encourage everyone to participate, and I may prompt you to contribute if necessary.
2. There are no wrong answers. Everyone’s experiences and opinions are valuable. Please express your views on the topics, whether you agree or disagree. We aim to gather a range of opinions.
3. Everything said in this group will remain anonymous.
4. We will record this session to extract as much information as possible from the discussion. However, no one will be identified in the reports, and the recordings will be destroyed after analysis.

**Icebreaker**

**Questions:**

1. Please tell us your name and how you would define medication adherence for Hypertension?
2. In your view, what are the main reasons patients forget to take their hypertension medication? (If necessary, to help facilitate the discussion, ask: "For those who take medication multiple times a day, does forgetting usually happen in the morning, at night, or both?")
3. What external or internal factors might contribute to patients not taking their hypertension medication as prescribed?
4. What are the main side effects that patients experience or report when they start taking medication to manage hypertension?
5. How do you think these side effects could be improved or avoided?
6. Is there any advice that could make it easier for patients to take their hypertension medication?
7. In your opinion, could there be any strategies that might help patients adhere to their hypertension medication?
8. In your opinion, what role does the family play in the treatment of hypertension, and how could this role be optimised?
9. In terms of healthcare provision, particularly at Health Primary Care Centres or Pharmacies, what could be done to help improve medication adherence for hypertension?
10. Do you think the use of technology (such as mobile phones, computers, or other devices) could help patients take their hypertension medication? If so, how?
11. Is there anything else you would like to add regarding what has been discussed or anything you feel is important to mention?

**Session Closure:**
A summary of the group's discussion will be presented, followed by a formal thank you to each participant for their contribution.
